# Deep learning-enabled fluorescence imaging for oral cancer margin classification in preclinical models

**DOI:** 10.1117/1.JBO.30.S3.S34109

**Published:** 2025-09-12

**Authors:** Hikaru Kurosawa, Natalie J. Won, Jack B. Wunder, Sujit Patil, Mandolin Bartling, Esmat Najjar, Sharon Tzelnick, Brian C. Wilson, Jonathan C. Irish, Michael J. Daly

**Affiliations:** a University Health Network, Princess Margaret Cancer Centre, Toronto, Ontario, Canada; b University of Toronto, Department of Otolaryngology-Head and Neck Surgery, Toronto, Ontario, Canada; c University of Toronto, Department of Medical Biophysics, Toronto, Ontario, Canada

**Keywords:** molecular guided surgery, depth-resolved fluorescence imaging, spatial frequency domain imaging, deep learning, oral cancer surgery

## Abstract

**Significance:**

Oral cancer surgery demands precise margin delineation to ensure complete tumor resection (healthy tissue margin >5  mm) while preserving postoperative functionality. Inadequate margins most frequently occur at the deep surgical margins, where tumors are located beneath the tissue surface; however, current fluorescent optical imaging systems are limited by their inability to quantify subsurface structures. Combining structured light techniques with deep learning may enable intraoperative margin assessment of 3D surgical specimens.

**Aim:**

A deep learning (DL)-enabled spatial frequency domain imaging (SFDI) system is investigated to provide subsurface depth quantification of fluorescent inclusions.

**Approach:**

A diffusion theory-based numerical simulation of SFDI was used to generate synthetic images for DL training. ResNet and U-Net convolutional neural networks were developed to predict margin distance (subsurface depth) and fluorophore concentration from fluorescence images and optical property maps. Validation was conducted using *in silico* SFDI images of composite spherical harmonics, as well as simulated and phantom datasets of patient-derived tongue tumor shapes. Further testing was done in *ex vivo* animal tissue with fluorescent inclusions.

**Results:**

For oral cancer optical properties, the U-Net DL model predicted the overall depth, concentration, and closest depth with errors of 1.43±1.84  mm, 2.26±1.63  μg/ml, and 0.33±0.31  mm, respectively, using *in silico* patient-derived tongue shapes with closest depths below 10 mm. In PpIX fluorescent phantoms of inclusion depths up to 8 mm, the closest subsurface depth was predicted with an error of 0.57±0.38  mm. For *ex vivo* tissue, the closest distance to the fluorescent inclusions with depths up to 6 mm was predicted with an error of 0.59±0.53  mm.

**Conclusions:**

A DL-enabled SFDI system trained with *in silico* images demonstrates promise in providing margin assessment of oral cancer tumors.

## 1 Introduction

Oral cancer surgery exhibits high rates of inadequate margins relative to other common solid tumors.^1^ Recent clinical studies report inadequate margin rates of over 30%,^2^ which have a direct negative impact on patient outcomes.^3^^–^^5^ In the oral cavity, as per the National Comprehensive Cancer Network, the closest margin distance—distance from the tumor to the specimen surface—of <1  mm is defined as positive, 1 to 5 mm as close, and >5  mm as clear.^6^^,^^7^ A key challenge in oral cancer surgery is determining the extent of tumor infiltration into surrounding tissue, as more than 87% of inadequate (positive and close) margins occur in these cases.^8^ Most oral cancers originate on a mucosal surface (e.g., tongue), which may provide visual cues to help delineate the lateral extension of disease prior to resection. When assessing tumor depth, however, surgeons must rely on preoperative imaging and intraoperative palpation, both of which can lack precision.^9^ The challenges of intraoperative surgical margin assessment, particularly along the deep resection surface, present the need for improved intraoperative techniques.

Recent clinical trials in fluorescence-guided oral cancer surgery provide promising results for resection guidance and margin assessment using tumor-specific contrast agents.^10^^–^^14^ These trials also provide insight into key limiting factors that motivate additional research. For margin assessment in resected specimens, de Wit et al.^13^ showed decreased accuracy across margin thickness, with area under the curve (AUC) values of 0.95 for tumor-positive (<1  mm), 0.78 for close margins (1 to 3 mm), and 0.65 for close margins (3 to 5 mm). Although near-infrared (NIR) fluorophores (650 to 950 nm) provide qualitative subsurface visualization due to low absorption within the biological imaging window,^15^ a quantitative, depth-resolved imaging system may offer improvement.

Several studies have investigated the use of fluorescence molecular imaging devices to resolve subsurface fluorescent structures. Rounds et al.^16^ demonstrated a dual-aperture fluorescence ratio approach to classify surgical margins of oral squamous cell carcinoma resections in an initial patient group. Multiwavelength approaches have been explored to predict the depths and concentrations of subsurface objects,^17^^–^^19^ by exploiting penetration depth variations due to changes in optical properties across the excitation and/or emission band. Spatial frequency domain imaging (SFDI) systems, which provide depth sensitivity across spatial frequencies,^20^^,^^21^ have also been investigated for subsurface depth quantification in preclinical models.^18^^,^^22^^,^^23^ In contrast to multiwavelength approaches, SFDI necessitates the use of a structured light source, making instrumentation more complex, but it avoids the dependence on optical property variations across fluorophore bands. Given these tradeoffs, multiwavelength and SFDI may be complementary (e.g., multiwavelength SFDI may offer potential improvements over the single-wavelength approach we present here). In prior SFDI work, Sibai et al.^22^ utilized the decay rate of the modulation amplitude of the fluorescence signal to calculate subsurface fluorophore depth, modeling the object as a point fluorescent source. The algorithm demonstrated depth recovery up to sub-surface depths of 9.5 mm in brain-like optical properties. Smith et al.^23^ proposed a deep learning (DL) driven method that coupled the use of fluorescent lifetime imaging and SFDI-based optical property maps to predict the depth of fluorescent inclusions. In both studies, however, the algorithm validation was limited to simple inclusion shapes such as a cylindrical glass capillary, and the system capacity in more complex scenarios is not explored. Specifically, our group aims to develop an SFDI system that can predict subsurface depths of complex tumor shapes encountered in oral cancer cases.

Here, we build upon our previous work in fluorescence depth imaging for oral cancer surgery by applying a DL-enabled SFDI system (SFDI-DL) to preclinical models of margin assessment. Our earlier work by Won et al.^24^ demonstrated the utility of this technology to quantify the depth of the bottom surface of infiltrative tumors. This assumed an *in vivo* imaging scenario of the mucosal surface of oral cancer. In this study, we explore the application of SFDI-DL in a post-resection scenario, assessing surgical margins of the basal surface of the *ex vivo* surgical specimen. We focus on imaging the deep surface of the specimen as it is most often involved in the presence of positive and close surgical margins.^7^ In our previous work, we adopted the Siamese convolutional neural network (CNN) architecture, using spatial frequency fluorescence images and optical property maps as inputs and tumor thickness and concentration as outputs.^24^ The inputs were encoded using a ResNet structure, which is characterized by the use of residual blocks that include identity shortcut connections, facilitating information from previous layers to be carried forward.^25^ This relatively simple architecture performed well for *in silico* and phantom test cases of infiltrative scenarios. With tumors originating at the surface, sufficient signal was obtained at every spatial frequency. However, in a surgical margin assessment scenario, less fluorescence signals are present at higher spatial frequencies, which sample only the shallow regions. This necessitates the extraction of relevant features from fewer spatial frequency images (i.e., low spatial frequency), and our previous ResNet model with a limited number of learned parameters may not be suited for this task. Therefore, the use of a deeper model architecture, U-Net, is explored in this paper, as it is commonly used in biomedical dense prediction tasks.^26^ In general, computational power not only scales with the number of parameters and the depth of the architecture but also improves performance. The ResNet architecture retains the same lateral dimensions throughout the entire path, whereas the U-Net structure condenses the lateral dimensions of the feature maps in the intermediate encoder path. This allows a deeper model such as U-Net to be trained even with similar graphics processing unit (GPU) memory demands as in ResNet. Here, the performance of the ResNet and U-Net models was compared to evaluate the potential benefits of the deeper U-Net model in learning subsurface depth quantification. Protoporphyrin IX (PpIX) is used as a model fluorophore with red illumination (630 nm); in principle, the algorithms are generalizable to other NIR fluorophores (e.g., IRdye800CW). This paper first reports on updates to the DL architecture and *in silico* training approach, followed by evaluations in simulations, phantoms, and *ex vivo* animal tissue models.

## 2 Methods

### 2.1 Deep Learning Architectures

**Fig. 1:**
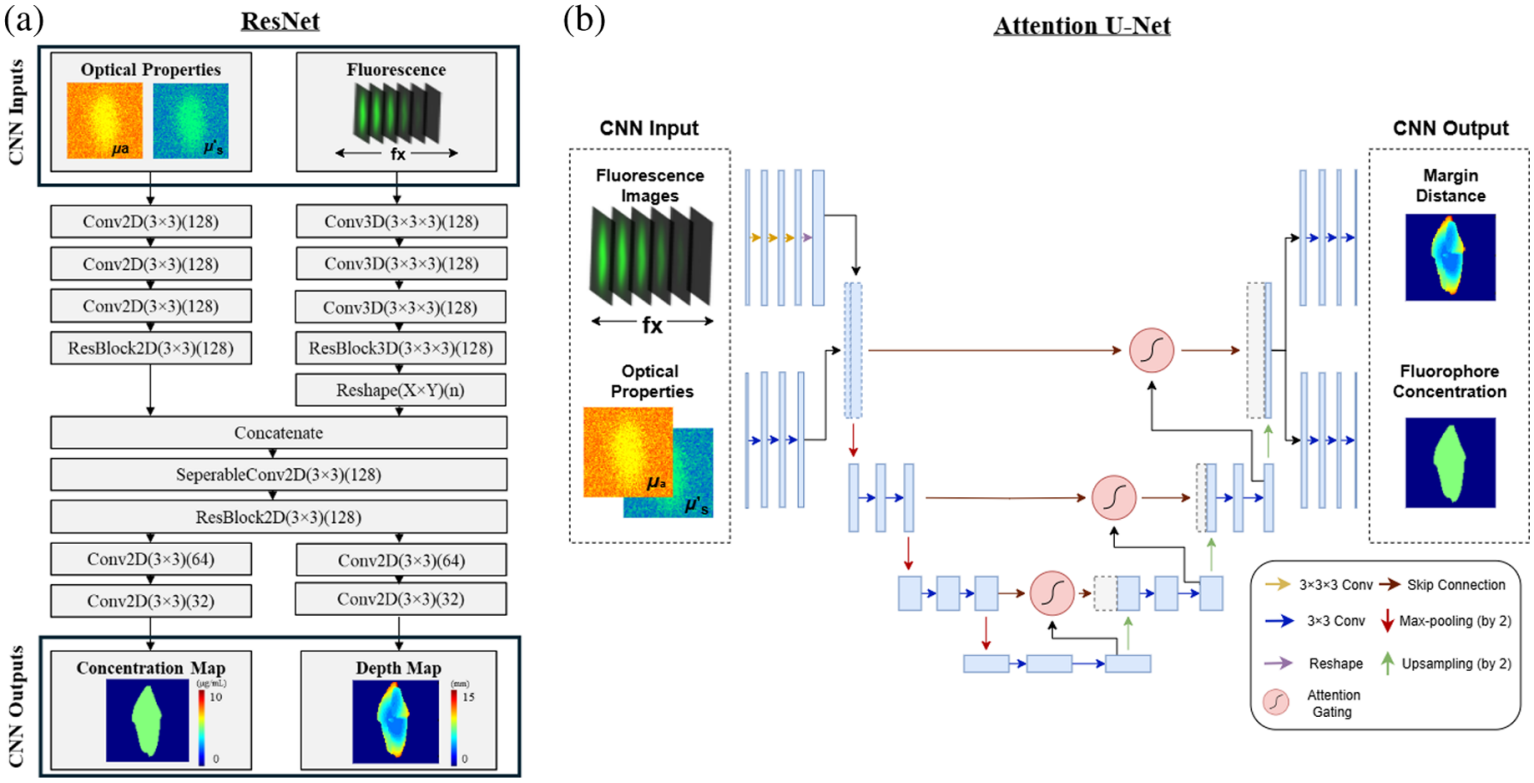
(a) ResNet and (b) Attention U-Net architecture diagram with fluorescence images across spatial frequency ($f_{x}$) and optical properties ($\mu_{a}$ and $\mu_{s}^{\prime }$) as inputs to predict the margin distance (subsurface depth of fluorescent inclusion) and fluorophore concentration as 2D maps.

Our previous study used the Siamese ResNet CNN to predict the thickness and fluorophore concentration of infiltrative oral cancer tumor models originating at the surface.^24^ The architecture is displayed in Fig. 1(a) (2,000,642 parameters), which we refer to as ResNet. In this study, we extend upon this previous work and also adopt the attention U-Net architecture, as shown in Fig. 1(b) (33,145,143 parameters), which we refer to as U-Net. The two models primarily differed in their method of encoding in the intermediate layers, in which ResNet used a residual block while U-Net used max-pooling layers.^25^^,^^26^

In ResNet, the fluorescence images acquired at six different spatial frequencies and optical property maps (absorption and reduced scattering) were processed separately through convolutional layers. Fluorescence images were processed using 3D filters to extract spatial information laterally (x and y axes) and in depth (across spatial frequencies), whereas optical property maps were processed using 2D filters. Residual blocks were used before and after concatenation of the two branches to encode relevant features. The extracted feature maps went through two separate 2D convolution branches to output the predicted fluorophore concentration and depth to the top surface of a buried fluorescent object.

In U-Net, similar to ResNet, the fluorescence images and optical property maps were processed in separate branches using 3D CNN and 2D CNN, respectively. The two branches were concatenated and underwent an encoder–decoder structure to extract image features at different image scales.^26^ Max pooling layers were used to reduce the dimensionality of the feature maps and preserve the most useful information for further processing.^27^ Upsampling layers were included to recover the resolution of the feature maps. In between the encoder and decoder paths, long skip connections from the encoder path were passed through the attention gates to disregard irrelevant information and support the decoding process.^28^ Similar to ResNet, the extracted feature maps were processed using two separate 2D convolution branches to predict the fluorophore concentration and subsurface fluorescent depth.

In both models, the subsurface depth of the fluorescent object measured along the imaging axis is defined as the margin distance. Each convolutional layer was followed by a ReLU activation function to introduce nonlinearity. A separate model, with a dropout layer after each convolutional layer before concatenation, was also trained specifically for testing on phantom and *ex vivo* datasets to provide robustness against overfitting.^29^

### 2.2 Deep Learning Training

**Fig. 2:**
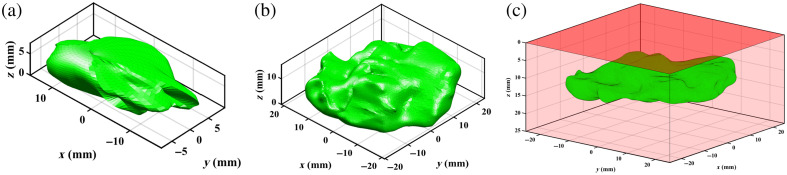
3D tumor shape models used for deep learning testing. (a) Composite spherical harmonics and (b) MRI-contoured patient tongue tumor shape. (c) Illustration of a tumor (green) submerged inside healthy tissue (red) with the deep surface oriented upwards.

The DL architectures were trained with *in silico* data using a synthetic tumor shape model based on spherical harmonics and an in-house numerical diffusion theory light propagation model, described in detail previously.^24^ Here, 10,000 synthetic tumor shapes were generated using composite spherical harmonics (CSH) in MATLAB. CSH were created by merging four spherical harmonics with the following randomized parameters: order and degree between 2 and 6, width between 5 and 40 mm, height between 5 and 10 mm, and closest top surface depth between 1 and 10 mm. An example CSH shape is displayed in Fig. 2(a). These shapes were inputted into the numerical light propagation model to produce synthetic reflectance and fluorescence images (101×101  pixels, 0.5  mm/pixel) across six spatial frequencies ($f_{x}=[0,0.05,0.1,0.15,0.2,0.25]\;{\mathrm{mm}}^{-1}$). Simulation time was ∼1 h. Reflectance images were further processed to estimate absorption and scattering coefficient maps using SFDI lookup tables computed at two spatial frequencies ($f_{x}=0$ and $0.2\;{\mathrm{mm}}^{-1}$).^20^ The training set was generated for an excitation wavelength of 630 nm, with randomly assigned homogeneous optical properties in each tissue: absorption $\mu_{a}$ between 0.0015 and $0.015\;{\mathrm{mm}}^{-1}$, scattering $\mu_{s}^{\prime }$ between 0.75 and $2\;{\mathrm{mm}}^{-1}$, and protoporphyrin IX (PpIX) fluorophore concentration between 1 and 10  μg/ml with quantum efficiency (η) of 0.046 per tumor case.^30^ This absorption range corresponded to a total hemoglobin (THb) concentration between 0.5 and 5  g/L and oxygen saturation of 95%. In addition, a randomized amount of fluorescence was added to the healthy tissue background, ranging from 0.1% to 50% of the tumor fluorescence concentration. For testing on *ex vivo* animal tissue with lower scattering than typical human tissue, a second training set was generated with absorption $\mu_{a}$ between 0.01 and $0.3\;{\mathrm{mm}}^{-1}$ and scattering $\mu_{s}^{\prime }$ between 0.3 and $0.7\;{\mathrm{mm}}^{-1}$ to ensure coverage of the observed optical properties within the training range. All data was inputted into the DL model on Amazon Web Services SageMaker with an ml.g5.2xlarge instance type (1 NVIDIA A10G GPU, 8 vCPU) for ∼2  h of training per data set. A depth of 10 mm was assigned to all background points to ensure the closest distance in the depth map corresponded to the tumor body rather than healthy tissue.

### 2.3 *In Silico* Testing

For *in silico* testing, 1000 CSH tumor shapes were newly generated with the same parameter ranges as the training set. The test cases had fixed optical properties ($\mu_{a}=0.0045\;{\mathrm{mm}}^{-1}$, $\mu_{s}^{\prime }=1\;{\mathrm{mm}}^{-1}$), fluorophore concentration (5  μg/ml), and randomized healthy tissue fluorescence between 0.1% and 50% of the tumor fluorophore concentration. These values fall within nominal ranges for both healthy oral tissue and cancer.^31^ To evaluate the capacity of SFDI-DL to make predictions for tumor shapes with complex features representative of real tumors, oral cancer tumor meshes were extracted by contouring preoperative MRI images of tongue cancer patients as displayed in Fig. 2(b). This was performed under institutional ethics board approval for retrospective patient data access (University Health Network REB #22-5471). For all 20 patient-derived data, the deep surface (at time of resection) was oriented upwards, and a layer of background tissue was created atop with a minimum margin distance of 1 to 10 mm at 1 mm intervals, for a total of 200 datasets. This orientation is illustrated in Fig. 2(c).

### 2.4 SFDI Instrumentation

A prototype SFDI system has been developed for imaging. Further details of the system are detailed in previous work;^24^ briefly, the system combines a high-resolution projector (DLi6500 1080p Optics Bundle, DLi, Austin, Texas, United States), 14-bit monochrome charge-coupled device (CCD) camera (Pixelfly USB, PCO AG, Kelheim, Germany), light-emitting diode (LED) light engine (Spectra X, Lumencor, Beaverton, Oregon, United States), 675-nm-long pass filter (ET700/75m, Chroma Bellows Falls, VT, United States), and 6-position motorized filter wheel (88-171, Edmund Optics, Barrington, NJ, United States).

In phantom and *ex vivo* experiments, reflectance and fluorescence image sets were acquired at six spatial frequencies ($f_{x}=[0,0.05,0.1,0.15,0.2,0.25]\;{\mathrm{mm}}^{-1}$) and three phase shifts ([0 deg, 120 deg, and 240 deg]), totaling 36 images (18 reflectance and 18 fluorescence). Nominal acquisition times for each reflectance and fluorescence image were 24 and 4000 ms, respectively. The camera lens aperture was set to an f/# of 2.8.

### 2.5 Phantom Testing

**Fig. 3:**
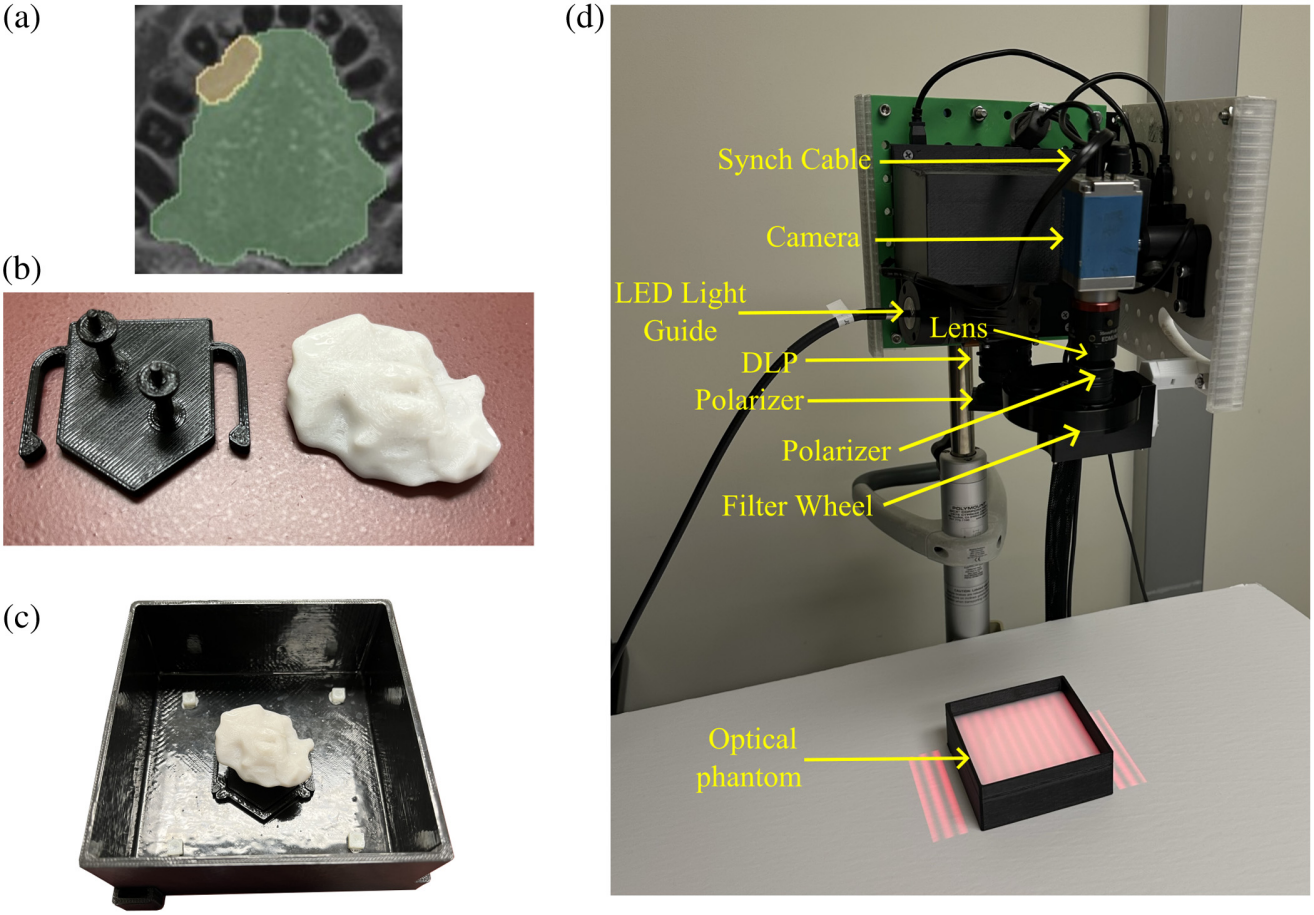
(a) MRI-contoured patient-derived oral cancer tumor. (b) Fluorescent agar phantom (right) created using the contour and a stage with circular pegs (left). The solid phantom was mounted onto (c) a mounted optical phantom fixed in a 3D-printed container. (d) SFDI imaging liquid phantom.

Further testing was conducted with real SFDI images obtained using optical phantoms. The phantom design process is shown in Fig. 3. Contoured meshes of the patient-derived tumor shapes were 3D printed (Original i3 MK3, Prusa, Prague, Czech Republic) to create a silicone (Ecoflex 00-30, Smooth-On, Easton, PA) negative mold for agar (AGA.501, BioShop, Burlington, Canada) phantoms. A solid fluorescent agar phantom ($\mu_{a}=0.02\;{\mathrm{mm}}^{-1}$ and $\mu_{s}^{\prime }=1.4\;{\mathrm{mm}}^{-1}$) was created to represent the tumor region and a nonfluorescent liquid phantom without agar ($\mu_{a}=0.011\;{\mathrm{mm}}^{-1}$ and $\mu_{s}^{\prime }=1\;{\mathrm{mm}}^{-1}$) for the healthy tissue region. The solid and liquid phantoms were made to approximately match the optical properties of tumor and healthy oral tissues, respectively. India Ink (Higgins PBk7, Chartpak Inc, Leeds, MA) was used as the absorbing agent and Intralipid (Fresenius Kabi, Toronto, Canada) as the scatterer. For the solid tumor phantom, PpIX (P8293, Sigma-Aldrich, Burlington, MA) was included as the fluorophore (5  μg/ml) and iohexol (Omnipaque, GE Healthcare, Chicago, IL) as the contrast agent for computed tomography (CT) scanning (10  μg/ml). The tumor solution was poured into silicone negative molds, forming a solidified tumor phantom, as shown in Fig. 3(b). This solid phantom was mounted onto circular pegs of a 3D-printed stage with the basal surface (i.e., most infiltrative surface) of the tumor oriented upwards. The stage was fixed onto the base of a customized container as shown in Fig. 3(c). Both customized 3D-printed components were made from black polylactic acid (EconoFil Standard PLA Filament–1.75 mm, InkSmith, Kitchener, Canada) ($\mu_{a}=20\;{\mathrm{mm}}^{-1}$ and $\mu_{s}^{\prime }=0.1\;{\mathrm{mm}}^{-1}$). The background liquid phantom was poured into the container until the tumor’s top surface was covered. Using the known container volume, liquid background was added to vary the closest subsurface depth to heights of 2, 4, 6, and 8 mm. A fixed distance between the instrument and the liquid surface was maintained by lowering the adjustable stage by the same height as the added liquid. The prototype SFDI system used for data acquisition is shown in Fig. 3(d).

**Fig. 4:**
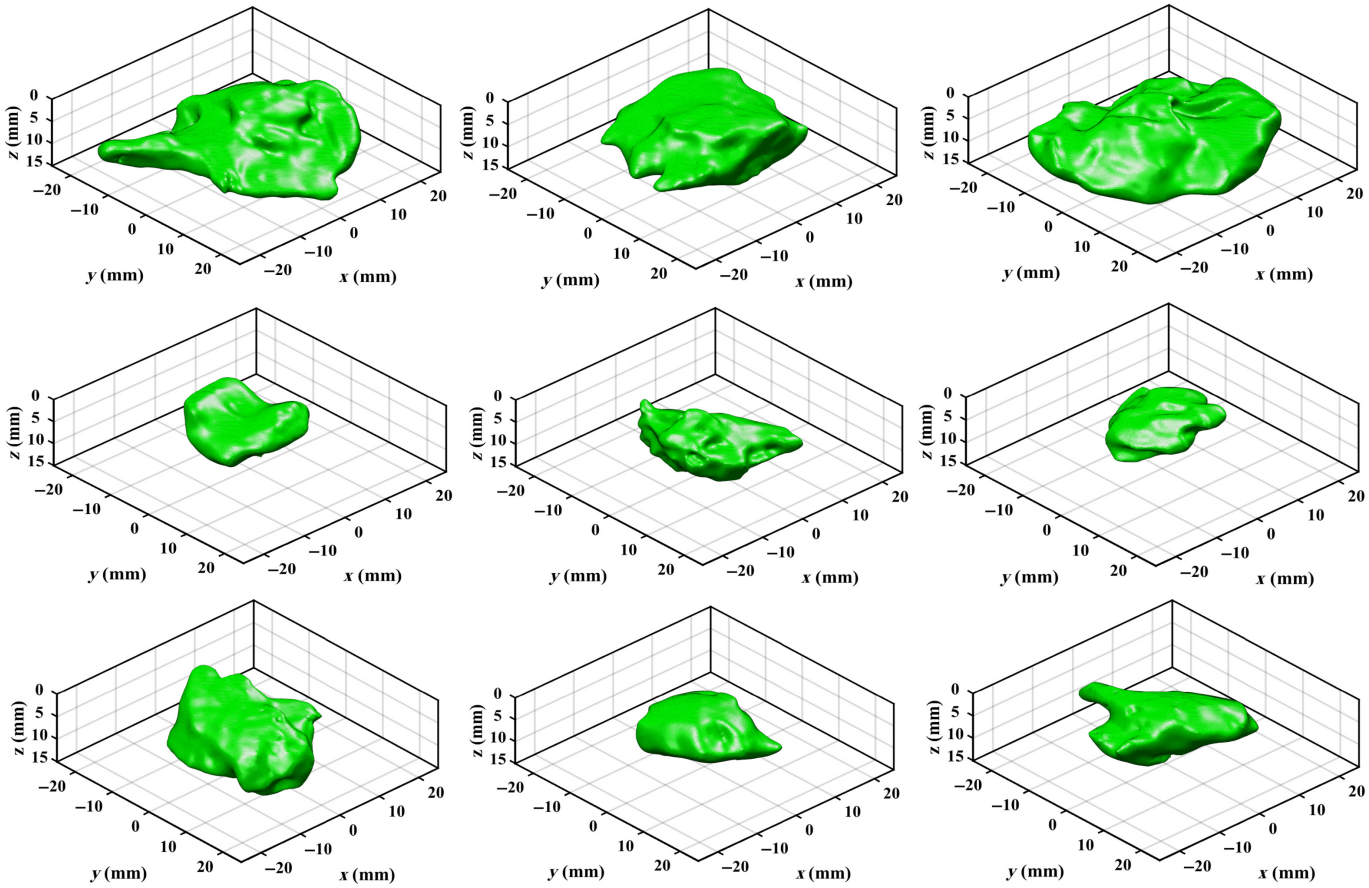
3D models of nine patient-derived tumor shapes used for phantom testing.

A representative set of nine out of the 20 patient-derived shapes was used for the phantom experiment, as shown in Fig. 4. Those shapes were selected to cover a range of widths (20 to 40 mm), thicknesses (5 to 15 mm), and topographies. To obtain the true geometry of the tumor phantoms, cone-beam CT scans (Siemens Cios Spin) of these solid phantoms were acquired. The CT scans were contoured in 3D Slicer software.^32^ Four fiducial points located inside the container were selected to map the orientation of the 3D mesh in the CT space onto the coordinates of the acquired SFDI images, and the transformed CT data were used to obtain the ground truth depth map. The true concentration map was obtained by assigning the known fluorophore concentration of 5  μg/ml to the tumor region.

### 2.6 *Ex Vivo* Testing

To validate with optically heterogeneous tissue, turkey breast was obtained from a local butcher and cut into rectangular blocks (∼70  mm width and length, ∼15  mm height). Six tissue blocks were prepared in total, each with nominal optical properties ($\mu_{a}=0.015\;{\mathrm{mm}}^{-1}$ and $\mu_{s}^{\prime }=0.45\;{\mathrm{mm}}^{-1}$) measured by the SFDI system. Semispherical molds were cut out at the center of these blocks to pour in agar solution for creating solid fluorescent tumor phantoms ($\mu_{a}=0.02\;{\mathrm{mm}}^{-1}$ and $\mu_{s}^{\prime }=1.4\;{\mathrm{mm}}^{-1}$ with 5  μg/mL PpIX). Variable thicknesses of tissue were preserved at the bottom surface of these molds to test different fluorescent inclusion heights. Once the phantom solidified, the tissue was flipped to mimic the margin assessment of infiltrative oral tumors in *ex vivo* specimens. The tumor shape contoured from the tissue phantom’s CT scan using 3D Slicer was manually aligned to match the agar phantom’s orientation during SFDI imaging. The same image processing steps done in 2.5 were applied and inputted to the prediction by the U-Net + dropout model. To extract ground truth margin thickness, 3D meshes for the turkey tissue and the agar phantom regions were extracted separately. Using MeshLab software,^33^ vertex-to-vertex distances between the tissue and tumor mesh were calculated to quantify the margin thickness at each point on the tissue mesh. Subsurface depths of the fluorescent inclusion in the tumor regions were further extracted from this map.

### 2.7 Analysis and Statistics

All analysis was conducted using Python 3.12.9.

For *in silico* test cases, the mean absolute error (MAE) and standard deviation (SD) were computed between the predicted and ground truth values of the depth map and fluorophore concentration maps over all pixels lying inside the tumor regions. MAE and SD at different depth ranges (0 to 5, 5 to 10, and 10 to 15 mm) were also computed. The minimum values of the depth map were then used to compute the mean minimum margin distance (closest subsurface depth) error. Linear correlation between the predicted and true closest subsurface depth values was also determined using sklearn 1.6.1.

For phantom and *ex vivo* testing, only the closest subsurface depth was considered for analysis because margin assessment is primarily concerned with the closest margin distance.^6^ To evaluate the effects of both a deeper model and dropout on depth prediction, ResNet and U-Net architectures with and without dropouts were compared. The MAE, SD, and linear correlation of the minimum margin distance were computed with evaluate system performance. Significance (*p<0.05, **p<0.01, ***p<0.001) was determined using an independent t test (scipy 1.15.2) based on the MAE and SD of each model.

For *ex vivo* testing, the U-Net with dropout model was used for prediction. MAE, SD, and linear correlation of the closest margin distance were calculated using the values obtained from the true depth map and the DL prediction.

## 3 Results

### 3.1 Simulation Results

**Fig. 5:**
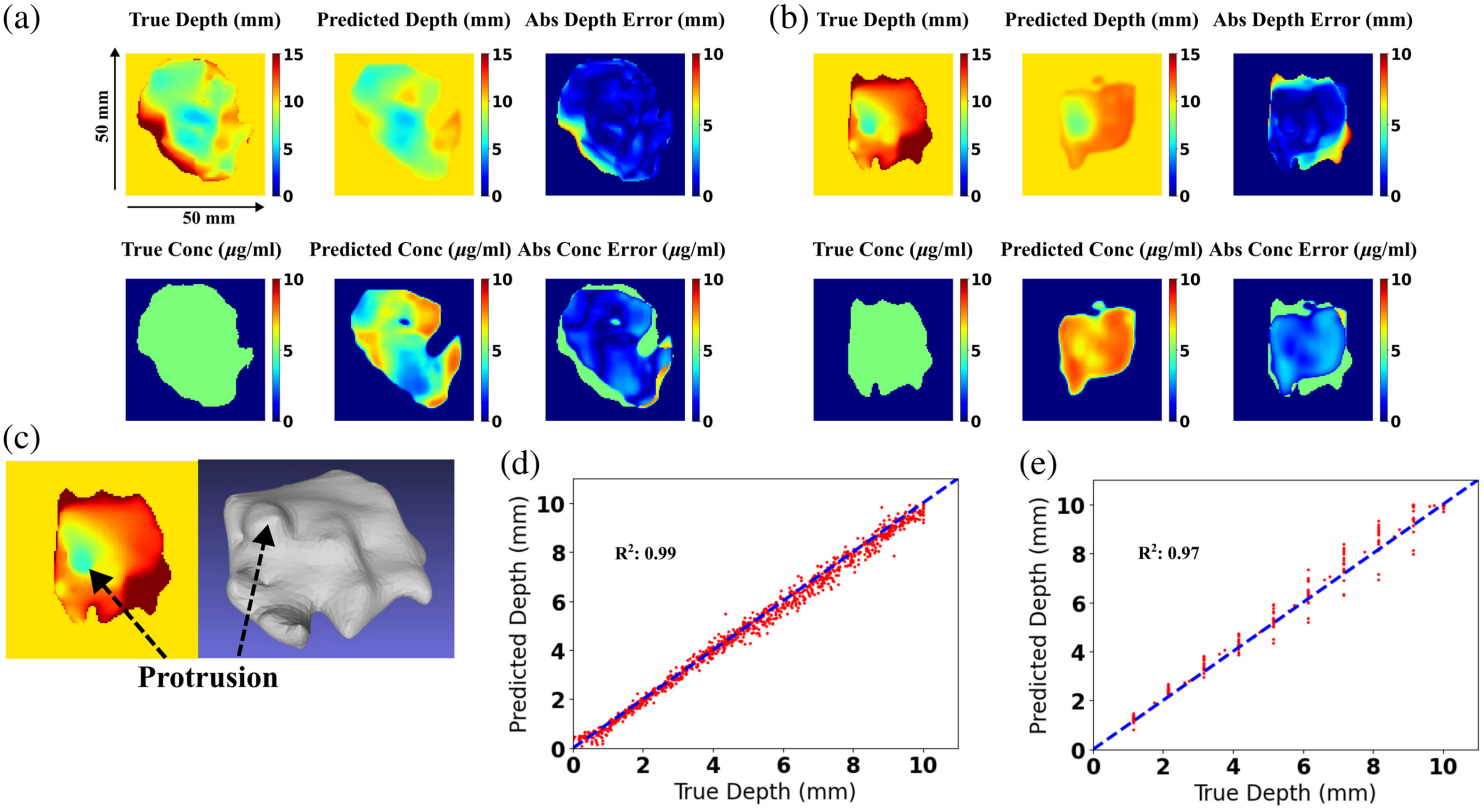
Depth and concentration map results for representative patient-derived tumor shape cases with (a) a minimum depth (margin thickness) of 2 mm and (b) a minimum depth 6 mm (c) 3D figure (right) of the tumor shape from (b) showing a protrusion (d) depth estimates of the minimum margin distance compared with the true depths for all composite spherical harmonics shapes (e) same for patient derived tumor shapes; the dashed line is the line of equality, and the best fit is indicated.

For *in silico* test cases, the DL predictions were generated using the U-Net model without dropout. For CSH, the subsurface depths and fluorophore concentration over all tumor regions were predicted with MAE of 0.41±0.75  mm and 1.41±1.24  μg/ml, respectively. In comparison, the patient-derived tumor shapes had higher subsurface and concentration errors of 1.43±1.84  mm and 2.26±1.63  μg/ml. The prediction and ground truth for the depth and concentration maps for a representative patient-derived tumor shape at shallow depth (closest distance = 2 mm) and deep depth (closest distance = 6 mm) are displayed in Figs. 5(a) and 5(b), respectively. Patient-derived tumor shapes contained greater irregularity in topography with complex features such as a protrusion structure seen in Fig. 5(c). The model predicted depth and concentration with minimal error for most points, but performance declined primarily in deeper regions (subsurface depth below ∼10  mm) as seen from Table 1.

**Table 1:** MAE and SD at different depth ranges for CSH and patient-derived tumor shapes.

Tumor shape model	Depths: 0 to 5 mm	Depths: 5 to 10 mm	Depths: 10 to 15 mm
CSH	0.31 ± 0.86	0.42 ± 0.62	0.73 ± 0.75
Patient-derived	0.35 ± 0.74	0.69 ± 0.73	1.63 ± 1.25

When error calculations are limited to the closest subsurface depth, which is most relevant for margin classification, the model predicted it with a mean minimum depth error of 0.19±0.17  mm for CSH cases and 0.33±0.31  mm for patient-derived tumor shapes. The minimum margin distance was predicted more accurately than the overall depth, with approximately one-half the error for CSH cases and one-quarter for patient-derived cases. As a result, both cases displayed strong agreement between the true and predicted closest margin depths, with $R^{2}=0.99$ for CSH [Fig. 5(d)] and $R^{2}=0.97$ for patient shapes [Fig. 5(e)]. Given the primary application of the model for predicting the closest margin distance, subsequent analysis focuses only on the evaluation of minimum depth errors.

### 3.2 Phantom Results

**Fig. 6:**
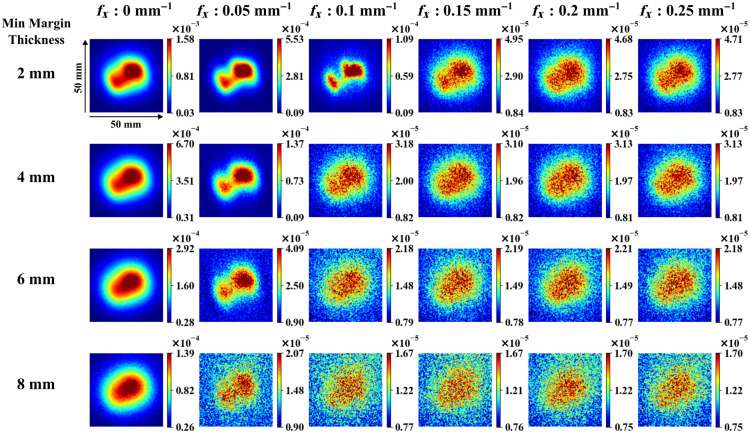
Diffuse fluorescence images at six spatial frequencies from different depths in the liquid phantom. Color bars correspond to unitless diffuse fluorescence values.

**Fig. 7:**
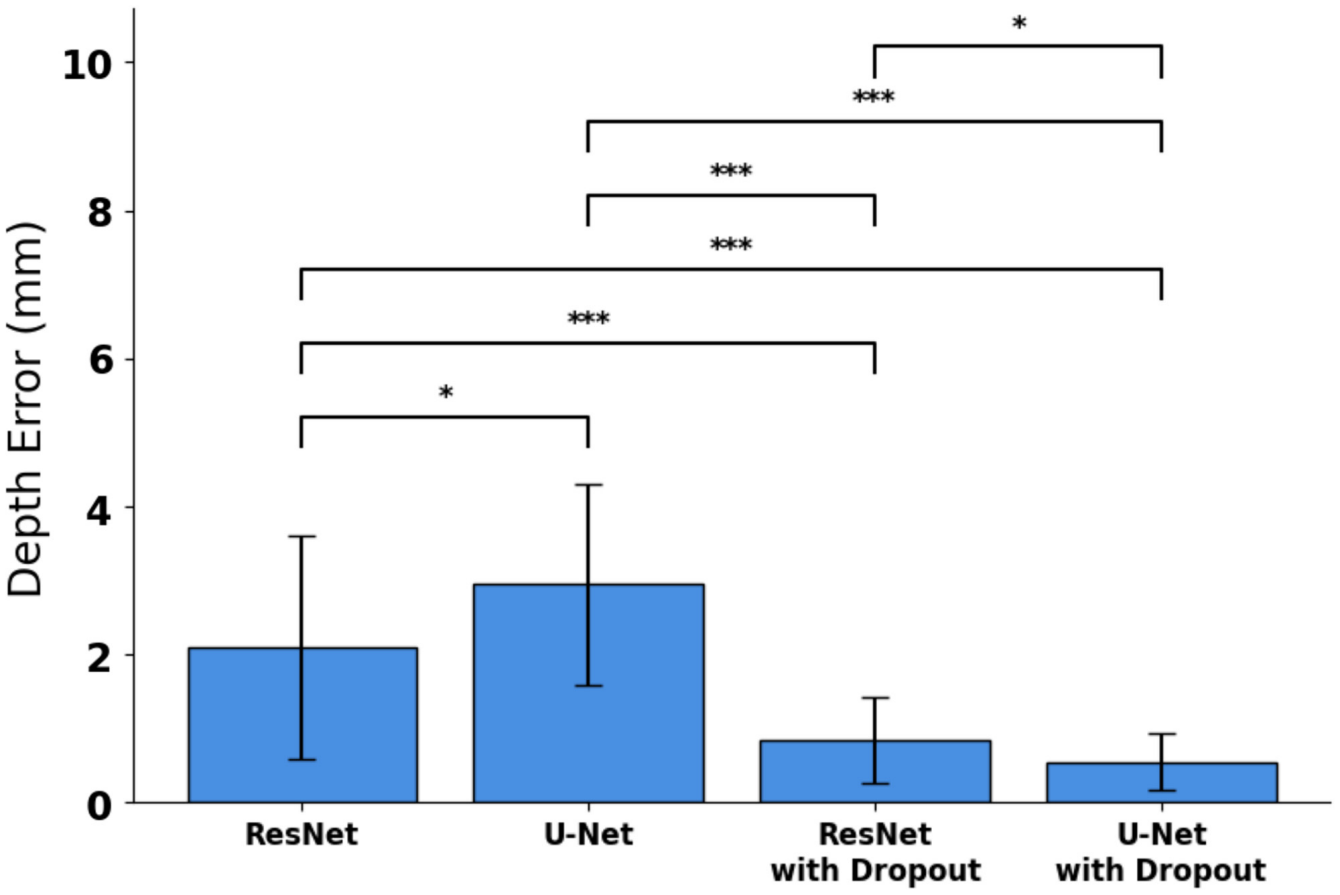
Mean closest margin distance error with SD for performance comparison in ResNet and U-Net models with and without dropouts. Statistical significance indicated (*p<0.05, ***p<0.001).

To illustrate how SFDI enables depth sampling, Fig. 6 displays fluorescence intensities for a single tumour shape across spatial frequencies of 0, 0.05, 0.1, 0.15, 0.2, and $0.25\;{\mathrm{mm}}^{-1}$ at closest subsurface depths of 2, 4, 6, and 8 mm. As expected, as the inclusion got deeper, measured fluorescence intensity decreased, especially at the lowest spatial frequency. The average minimum depth error calculated using the four DL models, tested on nine tumor shapes at four depths each, is shown in Fig. 7. ResNet and U-Net models without dropout predicted the closest distance with errors of 2.10±1.50  mm ($R^{2}=-0.33$) and 2.95±1.36  mm ($R^{2}=-1.1$), respectively. When dropout was applied, ResNet and U-Net models predicted with an average error of 0.85±0.58  mm ($R^{2}=0.79$) and 0.55±0.38  mm ($R^{2}=0.91$), respectively. The U-Net model with dropout attained significant improvement over the rest of the models (p<0.05), and therefore, this model was used for subsequent testing.

**Fig. 8:**
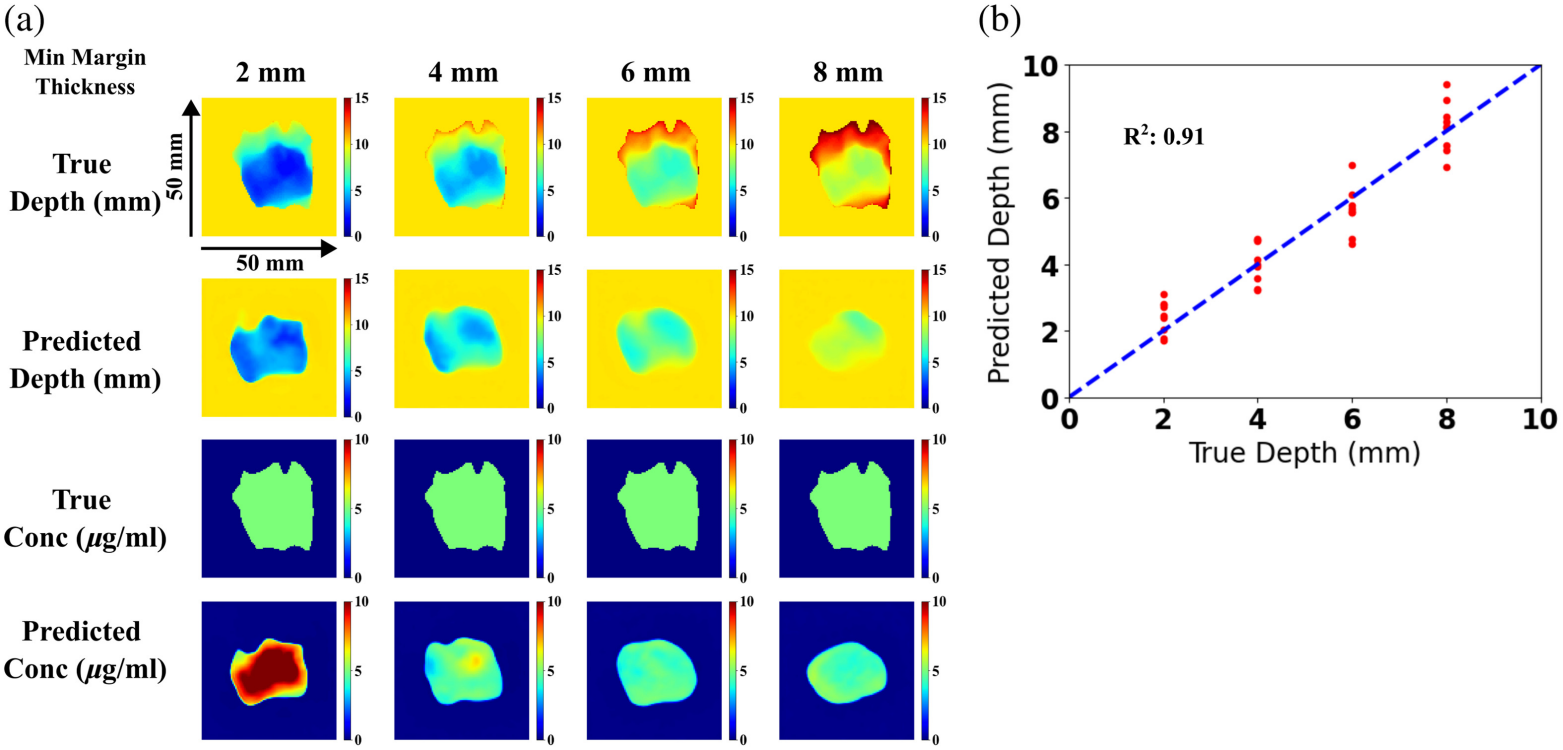
(a) Predicted and true depth and concentration maps at different depths using the U-Net model with dropout. (b) Minimum margin distance estimate compared with known true depth (line of equality shown with dashed line and corresponding best fit is indicated).

Predicted depth and concentration maps from the U-Net + dropout model are compared with ground truth across all four depths for a representative tumor case, shown in Fig. 8(a). The minimum margin distance predictions for all nine tumor shapes at the four different depths are displayed in Fig. 8(b). Although the differences in minimum depth error across depths were insignificant, deeper depths (6 and 8 mm) exhibited greater error and SD compared with shallower depths (2 and 4 mm), with an error of 0.60±0.44  mm and 0.51±0.31  mm, respectively.

### 3.3 *Ex Vivo* Results

**Fig. 9:**
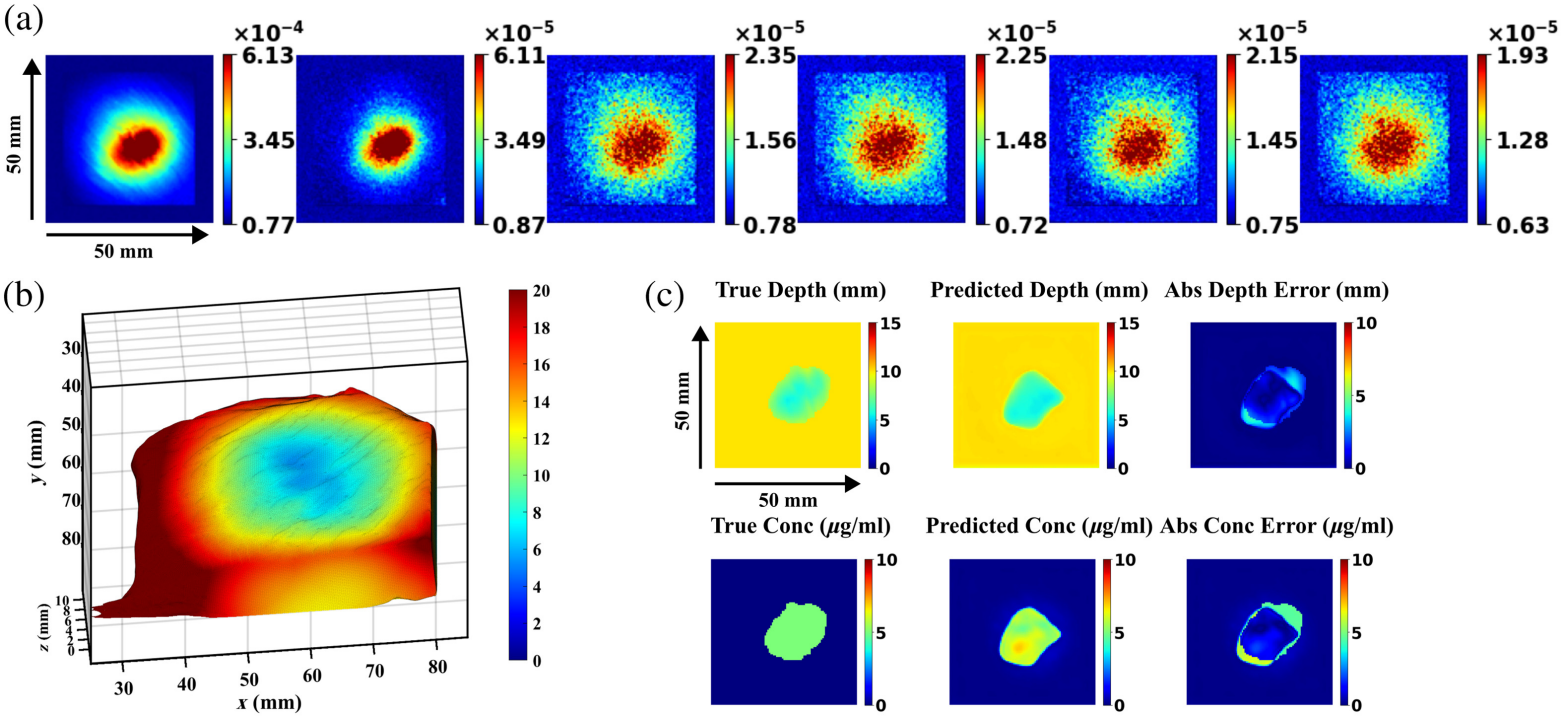
(a) Diffuse fluorescence images obtained at six different spatial frequencies of turkey meat with fluorescent inclusions (closest depth = 5.45 mm) beneath the tissue surface; regions outside the cropped area are padded with mean background intensity values. Color bars correspond to unitless diffuse fluorescence values. (b) 3D margin thickness surface rendering obtained using a CT scan. (c) True and predicted depth and concentration maps of an *ex vivo* tissue case using the U-Net + dropout model.

Fluorescence images of a representative tissue case with a fluorescent inclusion are displayed in Fig. 9(a). A relatively high signal to background ratio at lower spatial frequencies ($f_{x}=0$, $0.05\;{\mathrm{mm}}^{-1}$) is observed compared with the higher spatial frequencies ($f_{x}=0.1$, 0.15, 0.2, $0.25\;{\mathrm{mm}}^{-1}$). The 3D margin thickness graph for the same case, obtained from CT scan-derived meshes, is displayed in Fig. 9(b). The fluorescent inclusion was located at a subsurface depth of 5.45 mm. The true and predicted depth and concentration maps are displayed in Fig. 9(c), predicting the closest margin distance of 5.38 mm. Similarly, the closest subsurface depths of all cases are summarized in Table 2. For the six cases, the SFDI-DL system predicted the closest distance with MAE and SD of 0.59±0.53  mm.

**Table 2:** True and predicted closest subsurface depths for six different tissue cases with fluorescent inclusions.

	1	2	3	4	5	6
True closest depth (mm)	5.05	1.25	5.45	4.73	6.30	5.75
Predicted closest depth (mm)	4.98	0.16	5.38	5.34	4.84	5.53

## 4 Discussion and Conclusion

This study demonstrates the feasibility of an *in silico* trained DL-enabled SFDI system to quantify margin distance in preclinical oral cancer tumor models. It serves as an initial proof-of-concept that buried fluorescent objects with complex geometries, as observed in oral cancer resections, can be resolved for subsurface depth predictions. Within the optical property ranges observed in a nominal oral cancer environment,^31^ our SFDI-DL system detected the closest subsurface depth of a PpIX fluorescent inclusion with a mean error less than 0.6 mm for structures as deep as 10 mm in *in silico* test cases, 8 mm in phantoms, and 6 mm in *ex vivo* cases. This detection depth range is highly relevant to oral cancer surgery, where achieving a clear surgical margin of at least 5 mm is standard.^6^ Thus, the proposed SFDI system presents potential as an intraoperative margin assessment tool to provide more rapid surgical feedback without the need for specimen sectioning.

Margin distance prediction over all points was less accurate for more complex patient tumor shapes compared with synthetic CSH. This reduced performance can be attributed to the increased surface irregularities in patient tumor shapes (e.g., steeper surface, protrusions, asymmetry, concave geometries). It may also be due to the difference in population distribution between the training and testing data. As our DL model was trained using CSH, it learned the feature characteristics of CSH shapes but may not generalize well to the complexity of patient-derived tumors. However, this is not a major limitation in our application of margin assessment, where the clinical objective is to identify the closest distance from the tumor to the specimen surface. The system demonstrated better accuracy for detecting the shallowest point than for depth prediction across the entire tumor, which aligns well with the primary clinical need. For other surgical scenarios (e.g., glioma resection); however, the applicability of this technology may vary due to differences in clinical objectives and tissue environments. Different tissue environments present different optical properties, which directly influence the effective sampling depth.^17^^,^^21^ Conducting a prior investigation of the effective penetration depth range using simulation tools such as Monte Carlo may be beneficial in determining the suitability of the system to the desired application and conditions.

In this study, two DL architectures were compared with assess the utility of a deeper model for depth predictions. Although no significant difference in performance between the models was observed for *in silico* test cases, significance was attained when testing on real SFDI images. It is also important to note that this improvement was only possible with the inclusion of dropout layers at the beginning branch to perform well on the phantom data as seen in Fig. 7. DL aims to optimize for a nonconvex problem with multiple local minima. Training a single model on one dataset may converge toward a local minimum, which may lead to a suboptimal solution. A common machine learning solution that often yields better performance is model combination, which involves training multiple models with the same architecture and averaging the outputs at test time. However, training multiple DL models is computationally expensive. One technique that addresses this problem is dropout, which can be used at training time to approximate model combination. Hence, dropout layers are known to serve as regularizers that introduce noise to the inputs and make the system more robust against overfitting, and it presents potential in adjusting for the discrepancy between simulated and real SFDI images^29^^,^^34^; however, dropouts in convolutional layers reduce the spatial correlations of the images.^35^ The use of a deeper model such as U-Net may have allowed the system to accommodate this limitation, yielding improved performance compared with Res-Net with a dropout model. This final model predicted the closest subsurface depths in *ex vivo* models with similar accuracy as seen in the phantom experiment. This highlights the robustness of the system even when tested in heterogeneous tissue that resembles real biological environments.

Limitations in our study lie in the assumptions we have incorporated in the training and testing process. First, although diffusion theory-based simulations offer a computationally efficient approach to generating synthetic images, they rely on the assumption of isotropy. This is only valid when the scattering coefficient dominates the absorption coefficient.^36^ Monte Carlo simulations to better model light propagation are under development to help address this although they are much more computationally demanding. Second, our simulation used a simple shape model, CSH, for generating images. This shape model does not fully capture the complex nature of oral cancer tumor shapes such as irregular topography, protrusions, or the presence of “satellite buds.”^37^ More advanced shape modeling using mathematical expressions for cancer development or 3D generative AI may be beneficial.^38^^,^^39^ Third, the fluorophore was assumed to be confined to the tumor volume and homogeneously distributed throughout. *In vivo* fluorophores, however, preferentially accumulate at the tumor site but often exhibit heterogeneous distribution and can leak into the surrounding tissue environment. In addition, it is known that some fluorophores (e.g., nanoparticles) tend to accumulate at the tumor periphery due to tumor vasculature.^40^ Although an initial *in silico* investigation using a background fluorescent and peripheral fluorophore localization model did not lead to performance degradation, the extent of its impact in phantom and *ex vivo* cases remains uncertain. Fourth, as discussed in previous work,^24^ *in vivo* changes in fluorophore quantum efficiency may result in discrepancies with a trained model assuming a nominal value, which requires further investigation. Fifth, our phantom experiment design used a relatively small container (11×11×4  cm). Although a highly absorbent, low-scattering material was used to mitigate reflections from the container walls, some light scattering likely persisted. Monte Carlo simulations (data not shown) predicted less than a 10% difference in fluorescence at lower spatial frequencies between cases with and without the container. Nevertheless, the container introduces artifacts that may affect image accuracy. Optimal imaging conditions during phantom experiments and intraoperative scenarios should be further explored. Finally, the depth map outputs in this study quantified the subsurface depth of the fluorescent inclusion measured along the imaging axis. However, this may not necessarily correspond to the margin distance at each location. Imaging the same specimen from multiple angles may serve to provide several depth maps that may align closer with the true margin distance when used in combination. With a streamlined software pipeline, the entire data acquisition—imaging, processing, and depth estimation—is expected to take less than 2 min. Upgrading to a more sensitive camera (e.g., from the current CCD to a cooled sCMOS) would decrease the exposure times required for fluorescence imaging, which is currently the longest segment in the acquisition and processing pipeline.

In this study, our training set and test cases for *in silico* and phantom experiments assumed a homogenous optical property for the tumor and the background regions, along with a flat top surface. However, this represents a simplification of real patient cases with heterogeneity in optical properties and nonflat surface topography. Although the *ex vivo* model demonstrated system capacity in heterogeneous optical property environments with a nonflat top surface, further testing is required with more extensive optical and topography variations such as those found in patient specimens.^13^ For real resections, variation in the tissue optical properties through the volume can be expected with different amounts of blood perfusion and oxygen saturation, affecting the light penetration depth and signal quality.^31^ With a lower signal-to-background ratio, SFDI-DL performance is expected to be reduced, and future work will investigate the extent of the effect in higher absorption scenarios. Moreover, patient resections would only contain a limited amount of tissue at the sides. This creates a finite boundary to the space where light can propagate, which may introduce fluorescent artifacts into the image. Future work requires implementation in patient cases to assess the system’s sensitivity to these factors, as well as to compare SFDI-DL margin classification performance with standard clinical techniques and emerging optical methods.

## Data Availability

Available upon reasonable request to the corresponding author.
